# Supplementary material for: Catchment prioritization for freshwater mussel conservation in the Northeastern United States based on distribution modelling
Source: PLoS One. 2025 Sep 9;20(9):e0324387. doi: 10.1371/journal.pone.0324387 (PMC12419594; doi:10.1371/journal.pone.0324387)
Supplement: Attachment — Submitted filename: Reviewer response 2025 17 04.docx [file pone.0324387.s002.docx]

Dear reviewers,

Thank you for your careful review of our manuscript. The suggested edits have greatly improved the paper, and we hope we have addressed them satisfactorily. This paper is published through the USGS, so in addition to the revisions recommended by the reviewers, this draft also includes revisions in response to a USGS reviewer and the Bureau Approving Official. Below, I have included a table with individual responses to each reviewer’s comments as well as the USGS reviewer’s comments and responses.

We look forward to hearing any further feedback.

Sincerely,

Rebecca O’Brien

| **Reviewer comment** | **Line number (in document without track changes)** | **Revision** |
| --- | --- | --- |
| **Reviewer 1** |  |  |
| I would like to see some more applicability in the international context. At the moment, this is a regional study from the north-eastern United States | Throughout introduction and discussion | Thank you for this excellent suggestion. We added sections throughout the paper placing Northeastern U.S. mussels in the context of a broader global conservation efforts which both maked it better suited to PLOS One's audience and also improved the paper! |
| **Reviewer 2** |  |  |
| I would suggest modifying the title to reflect this (as it is the title may lead to narrowing down the range of readers), something like “Catchment prioritization for freshwater mussel conservation across the Northeastern United States using distribution modelling tools | 1 | Thank you for this suggestion. We changed the title to reflect the reviewer's much improved version. |
| 1) Having used data from since the 1950’, was the potential effect of outdated presence data been taken into account? If so, how? If not, this potential effect must be discussed further ahead. Some variables used have changed dramatically over the course of this time, so if they are used as explanatory the data will suffer from this shortcoming. | 149-153 | We added explanations of why we felt it was appropriate to include these older data, and insight into how the time was skewed towards newer records. We additionally added an explanation to the paper of how data was averaged across present and past records for landcover variables. |
| 2) Have the variables been used in the models as equivalent in importance and not weighted? From some sections of the text, it seems that there was some kind of variable weighting at some point, but this is not explained. | 235-236 | Thank you for pointing out the need for additional clarity here. We added a more detailed explanation of how regularization parameters can reduce the influence of certain variables. |
| 3) Was there no attempt to use bioclimatic variables? Why not? This is somewhat surprising as these may be used to predict future distributions, and this should be taken into account when prioritizing conservation actions. | 197-201 | We chose not to utilize dismo's bioclimatic variables because this enabled us to select variables that more closely reflected the aquatic habitats of our study (e.g., using stream temperature in place of air temperature) and it facilitated integration of this project with related modeling efforts. We added this explanation to the manuscript. |
| 4) Why were invasive species not used as potential explanatory variables? | 609-613 | Although zebra mussels and quagga are not a great concern in our study area, you are right that including the distribution of asian clam would have improved the study. We added a section to the discussion discussing how this could be a future direction. |
| 5) Methods for validating the models must be better accounted for in the methodology. | 273-285 | Thank you for pointing out the need to more clearly highlight this. Our discussion of model validation was buried in the methods, so we moved it to its own labled section to improve its visibility. We also added additional details about how model results were shared with experts to assess their accuracy in addition to the statistical assessments described in the text. |
| 6) Calculations for estimating prioritizations are difficult to replicate with the provided explanations alone. Maybe presenting an equation or example would help. | Table 3 | We apologize for the lack of clarity here. We added an example equation to the caption on table 3. |
| I feel that it would benefit from contracting the work with broader geographical scales. The interest to national-level conservation is briefly mentioned and should be extended (comparing with other species examples for instance). Comparison with other countries would also be relevant, especially considering there are quite a few published papers modelling freshwater mussel distribution. The impact of climate change should also be discussed, especially considering that no modelling of future distributions was made. | Throughout introduction and discussion | Thank you for this great comment. We added additional text explaining the place of American mussel biodiversity to the world as a whole and we added in previous research findings regarding drivers of mussel distribution. We also clarified in our discussion that we did incorporate climate change into our land protection model, and added additional references to other species and parts of the world. |
| 1) Replace “Study Geography” by “Study Area” | 126 | We made this change |
| 2) “Ample freshwater” (line 120) is a dubious expression. Better use “extensive river network”, large water bodies, etc. | 129 | We changed this phrasing to match the reviewer's suggestion. |
| 3) “level III Ecoregions” are referring to the EPA classification I presume. International reader will in general not understand what this refers to, so must be explained. | 132-133 | Thank you for catching the need to elaborate on this term. We have added an explanation of ecoregions. |
| 4) “These calculations” (line 278). Which calculations? Surely the ones to estimate catchment prioritization, but no calculations were presented at this point. | 316 | We clarified this language. |
| 5) Reintroductions: Although well explained, it would be better referring these concepts to the ones presented in the IUCN guidelines to reintroductions. | 311-312 and thorughout | Thank you for this excellent suggestion. We adjusted this language to reflect IUCN terminology throught the paper |
| 6) Table 5: Presents state letters instead of the actual names, without explaining in the legend. These are not broadly understood, so either write the names in full in the table or explain in the legend. | Table 5 | Thank you for catching this. We changed the state initials to state names |
| Finally, the short title does not seem short at all. Suggest: “Conservation prioritizing of freshwater mussels in Northeastern US” |  | Ha! Fair enough. This change will be made upon resubmission (done via submission website) |
| **USGS reviewer** |  |  |
| I think it is important to touch on what we do know, as presumably your SDMs will be informaed in part by prior knowledge. We actually know quite a bit about what governs mussel species distributions at regional and larger scales (e.g., biogeographic history, host fish occurrence/richness, disturbance regimes, thermal limits), but within streams, the relationships between mussels and their habitats can be harder to determine - although hydraulic habitat seems to be pretty universally important. | 85-90 | Thank you for this observation. We added additional text explaining some of the variables that have been identified as driving freshwater mussel distribution. Additionally, we added text and citations to the discussion clarifying that some predictors do not appear to translate across sites. |
| "In the Northeastern United States (U.S.), freshwater mussel conservation has generally consisted of state-by-state monitoring programs with uneven and uncoordinated survey efforts" References or examples? | 100-101 | We added the following citations: Raithel and Hartenstine 2006; Fichtel and Smith 1995; NHFGD 2015; Swartz and Nedeau 2007 |
| I think this is an important point to elaborate on. Patterns in mussel biodiversity are strongly structured by the hierarchically nested nature of catchments and sub-catchments. Pointing out that catchment boundaries cross political boundaries would be good here, as it highlights the usefulness of a regional framework (Rather than one constrained by state boundaries). | 102-104 | Thank you for this suggestion. We added text discussing how mussel distributions cross political boundaries and are influenced by the wider drainage conditions |
| Some information on the prevalence of these different data types would be useful, if only as a supplement. The types of surveys done are especially relevant. For example, semi-quantitative timed searches are better than quantitative surveys for detecting rare species in most cases. | 161-165 | This is a valid concern, but unfotunately, the majority of data did not include the type of survey method used, so we can't provide a reliable summary of methodology. Nonentheless, we added text to the paper acknowledging the limitations of including all survey types and further explaining why we did so. We were able to add details about the lentic/lotic split. |
| Here as well, more information on the distribution of the data would be useful as background (again, could be a supplement, but useful for someone who is versed in mussel ecology). | 170-176 | Thank you for this suggesteion. We added additional details on where some of the data was less precise. |
| I think describing the model structure/methods should come before the details on how the model outputs were interpreted. | 275-292 | This was an excellent suggestion. We moved this section of text to later in the methods and it improved the flow of the paper markedly. |
| I was a bit confused by this wording at first - so the level of observation is the catchment, and the correlation is between probability of occurrence in the species pairs across all catchments? Assuming that is correct, the wording should say “correlations among … and … across all catchments” or something similar (as opposed to “similarities”). | 289-292 | Sorry for the confusing wording on this! We changed language to "After modeling each species, we tested for correlations between each species pair in the predicted probability of occurrence across catchments using Spearman’s rank correlations (ρ) and the cor.test function in R’s stats package" |
| Some justification for the inclusion of these variables with reference to the literature would be appropriate in the introduction. I left a comment above suggesting that you add background on what we do know. I know Caryn Vaughn’s research group has done quite a bit of work on long term impacts of climate, impoundment, and land use impacts. I believe Dan Hornbach has been involved with some similar long-term work. | 85-90 | Thank you for this suggestion. We added the text to the introduction explaining some of the previously identified variables and here we explained why we did not use the bioclimatic variables commonly used in MaxEnt models. |
| What is the difference between “catchment” and “watershed”? The terms are generally interchangeable, and as far as I know, do not connote a difference in spatial scale. Use HUC codes to differentiate scale. | 171-191 | Thank you for highlighting the need for a clear definition here. We added the StreamCat-based definition for the terms catchment and watershed in the introduction. ("For the purposes of this paper, a catchment is defined as the area that drains directly to an NHD stream segment (excluding upstream contributions). A set of hydrologically-connected catchments upstream of and flowing into a focal catchment is referred to as a watershed (Hill et al., 2016)". In this location in particular, we eliminated the term watershed and replaced it with a catchment-based definition of this scale of analysis. |
| I’m not an expert on SDMs but at least for linear models variance inflation factors are considered to be more appropriate than standard correlation tests for detecting multicollinearity. Plus, Spearman doesn’t test for linearity, just a monotonic relationship because it uses ranks. Here’s a quick Google results that explains this a bit: https://quantifyinghealth.com/correlation-collinearity-multicollinearity/. VIFs can be implemented using the car package in R. | 212-214 | Thank you for making us aware of this preferred methodlogy! We confirmed the variables included in the model did not show multicollinearity using VIF and added the associated methods and results. |
| This should be in the introduction: "Freshwater mussels have a complex reproductive cycle whereby their larvae parasitize host fish, which they rely on for dispersal (Modesto et al., 2018). Freshwater mussels can be limited to a few host fish species (for host specialist mussels), or they can be highly variable (for host generalist mussels). " | 78-80 | This is an excellent suggestion. We moved this information to the introduction and kept only a small reference to it here. |
| For all three categories, a bit more of a conceptual explanation of why values were added, subtracted, summed, etc. would be useful. It’s a bit tough to follow the math being done in these sections. You could even consider presenting an equation for how each score was calculated instead of listing the variables in tabular format. | 337-339, Table 3 | Thank you for highlighting the need for clarity here. We added details to the survey priority section explaining why we added/subtracted values since this did not have any explanation. We also added an equation to the caption of table 3 to facilitate its interpretation |
| "We limited our analysis of catchment priority for reintroduction to species with an RSGCN rank of high or very high conservation concern (Table 1)" This is worth mentioning in the legend for table 3, as well. | Table 1 | We added this clarification to the caption |
| "We tested for correlations between the percent protected land and percent projected land use change as well as between percent protected land and species richness using cor.test in R’s stats package." Why? | 383-385 | Thank you for catching this error! We removed this mistakenly included text and added text explaining our testing for correlations between percent protected and land and protection priority (in the relevant paper section). |
| This appears to be a typo. | 429 | Our apologies for the confusion here. The first apparent p is a ρ... Quite easy to misread! |
| Cite the release here? | 434 | Good catch! We added this citation |
| You mean all states were similar? Slightly ambiguous. | 470 | Thank you for catching the ambiguity here. We clarified this language by replacing "they" with "all states" |
| This is a broad trend in mussel ecology, and also a potentially problematic one. When we survey for mussels we look for mussel aggregations/beds. However, there are some species (e.g., Lampsilis teres) that are adapted to life outside of aggregations, and so are sometimes considered rare, when they are actually quite common. This may or may not be relevant to your discussion since your analysis was done at the catchment level, however, it is probably based primarily on data from spatially defined mussel beds. | 538-540 | This is an interesting point and an important observation. We added a discussion of this limitation to the discussion. |
| This could be a good place to highlight the differences in knowledge about mussel habitat at different scales. | 586-587 | Thank you for helping us identify a good place to include this discussion. We added the following text to this part of the discussion: "and factors that predict occurrence in one area may not always do so in another (Strayer et al., 2008) " |
| Remind us why land protection is beneficial for mussels. Readers may not make the connection between landscape factors and a benthic aquatic organism. | 590-592 | We added the following text to this part of the discussion: "Land protection is valuable for freshwater mussels as land use can significantly influence water quality and, in turn, mussel species richness (Cao et al., 2013) " |
| Maybe I missed it, but I couldn’t find this citation in the references section. | 763-766 | Thank you for catching this omission. We added this (quite important!) citation |
| Would this be more informative as a metric of performance if given as a percentage? | Table 5 | Although we agree that a percentage might make most sense here, the paired column ("Number of possible new catchments with species") can not smoothly be displayed as a percentage. Given this, we decided to keep number here so that the two were the same format. |
